# Dynamics of White Matter Plasticity Underlying Working Memory Training: Multimodal Evidence from Diffusion MRI and Relaxometry

**DOI:** 10.1162/jocn_a_01127

**Published:** 2017-03-30

**Authors:** Claudia Metzler-Baddeley, Sonya Foley, Silvia de Santis, Cyril Charron, Adam Hampshire, Karen Caeyenberghs, Derek K. Jones

**Affiliations:** 1 Cardiff University, Brain Research Imaging Centre (CUBRIC); 2 University Miguel Hernandez, Alicante, Spain; 3 Imperial College London; 4 Australian Catholic University

## Abstract

Adaptive working memory (WM) training may lead to cognitive benefits that are associated with white matter plasticity in parietofrontal networks, but the underlying mechanisms remain poorly understood. We investigated white matter microstructural changes after adaptive WM training relative to a nonadaptive comparison group. Microstructural changes were studied in the superior longitudinal fasciculus, the main parietofrontal connection, and the cingulum bundle as a comparison pathway. MRI-based metrics were the myelin water fraction and longitudinal relaxation rate R_1_ from multicomponent relaxometry (captured with the mcDESPOT approach) as proxy metrics of myelin, the restricted volume fraction from the composite hindered and restricted model of diffusion as an estimate of axon morphology, and fractional anisotropy and radial diffusivity from diffusion tensor imaging. PCA was used for dimensionality reduction. Adaptive training was associated with benefits in a “WM capacity” component and increases in a microstructural component (increases in R_1_, restricted volume fraction, fractional anisotropy, and reduced radial diffusivity) that predominantly loaded on changes in the right dorsolateral superior longitudinal fasciculus and the left parahippocampal cingulum. In contrast, nonadaptive comparison activities were associated with the opposite pattern of reductions in WM capacity and microstructure. No group differences were observed for the myelin water fraction metric suggesting that R_1_ was a more sensitive “myelin” index. These results demonstrate task complexity and location-specific white matter microstructural changes that are consistent with tissue alterations underlying myelination in response to training.

## Introduction

Training schedules that adapt task difficulty to optimally challenge a trainee have been shown to maximize training benefits and plasticity (Metzler-Baddeley & Baddeley, 2009; Smith et al., 2009). For instance, working memory (WM) capacity, our ability to temporarily maintain and manipulate information (Baddeley & Hitch, 1974), can be enhanced with adaptive training (Melby-Lervåg & Hulme, 2013; Shipstead, Redick, & Engle, 2012; Morrison & Chein, 2011), and such WM benefits are associated with plastic changes in parietofrontal networks (Caeyenberghs, Metzler-Baddeley, Foley, & Jones, 2016; Metzler-Baddeley, Caeyenberghs, Foley, & Jones, 2016; Takeuchi, Taki, & Kawashima, 2010; Olesen, Westerberg, & Klingberg, 2004). The neural substrates underpinning such plastic changes, however, remain poorly understood.

Recently, we compared the effects of 2 months of adaptive WM training (Cogmed, 2012; Klingberg et al., 2005) with a nonadaptive comparison activity that trained the same tasks but on three item spans only. Subtle changes across a number of gray matter regions including increased cortical thickness in the right frontal cortex and increased volume of the left pallidum were associated with adaptive WM training, whereas reductions in cortical thickness in the right pars triangularis were associated with repeated unchallenging comparison activities (Metzler-Baddeley et al., 2016). Using graph theoretical analysis (GTA) of white matter microstructural metrics, we found improved global integration within the right parietofrontal network after adaptive WM training (Caeyenberghs et al., 2016). This increase in network global efficiency was best captured by MR relaxation rates, notably the longitudinal relaxation rate R_1_ and was positively correlated with WM benefits.

Although GTA is helpful in understanding the effects of training at the global network level, this method confounds differences in the connectivity profile with differences in the microstructure of those connections. Moreover, the global approach precludes the interrogation of individual “edges” in the graph. Thus, on the basis of GTA alone, it can be difficult to infer about the nature and location of microstructural alterations within the network, and subtle activity-related changes may be missed. The aim of this study was therefore to explore the neural substrates underpinning adaptive training-induced white matter plasticity on the local level within parietofrontal white matter of the superior longitudinal fasciculus (SLF).

**Figure 1:**
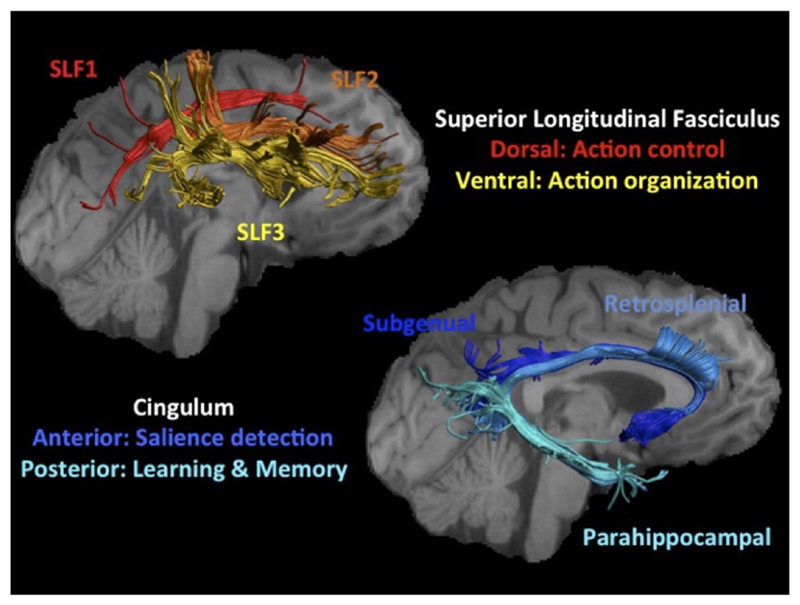
The top left hand visualizes the three subfascicles of the right SLF. The tracts were reconstructed for one participant and were displayed on their T_1_-weighted image visualizing the lateral view of the right hemisphere. The SLF1 (red) connects the dorsal-superior parietofrontal regions of the visual attention network proposed to be important for action control in WM (Rizzolatti & Matelli, 2003). SLF3 (yellow) connects ventral-inferior parietofrontal regions thought to be important for action organization and recognition (Rizzolatti & Matelli, 2003). SLF2 (orange) comprises central parietofrontal white matter that has been suggested to allow the cross-communication between dorsal and ventral visual attention networks (Thiebaut de Schotten et al., 2011). Parietofrontal cortical regions have been reported to undergo structural and functional changes after WM training (Takeuchi et al., 2011; Takeuchi, Sekiguchi, et al., 2010; McNab et al., 2009; Olesen et al., 2004). The lower right image displays the three subfascicles of the cingulum bundle (SGC in dark blue, RSC in blue, PHC in light blue) reconstructed for the same participant and displayed on a medial view of the right hemisphere. The cingulum bundle was chosen as comparison pathway for the SLF because SGC and RSC maintain projections to and from anterior salience network regions, which have been proposed to support WM action control by detecting salient stimuli in the environment (Dosenbach et al., 2008). The PHC forms part of the medial-temporal lobe network known to be important for learning and episodic memory.

The SLF is the largest intrahemispheric parietofrontal connection and comprises dorsal-superior (SLF1), central (SLF2), and ventral-inferior (SLF3) parietofrontal white matter (Thiebaut de Schotten et al., 2011; Makris et al., 2005; Figure 1). We expected plastic changes in the SLF because this bundle connects parietofrontal cortical regions that are known to be important for WM functions such as action control and organization (Dosenbach, Fair, Cohen, Schlaggar, & Petersen, 2008; Rizzolatti & Matelli, 2003) and have been shown to change with WM training (Takeuchi et al., 2011; Takeuchi, Sekiguchi, et al., 2010; McNab et al., 2009; Olesen et al., 2004). To assess the specificity of SLF changes, the subgenual (SGC), retrosplenial (RSC), and parahippocampal (PHC) portions of the cingulum bundle (Jones, Christiansen, Chapman, & Aggleton, 2013; Mufson & Mesulam, 1982) were also reconstructed as comparison pathways. SGC and RSC maintain anterior cingulate projections and form part of the salience network (Dosenbach et al., 2008), whereas PHC forms part of the extended medial-temporal lobe memory network (Jones, Christiansen, et al., 2013).

White matter plasticity is thought to be largely driven by axon myelination (Fields et al., 2014; Zatorre, Fields, & Johansen-Berg, 2012; Fields 2010), which regulates saltatory conduction and has been linked to neuronal activity (Gibson et al., 2014) and to the learning of new motor skills in animals (Fields et al., 2014; McKenzie et al., 2014; Sampaio-Baptista et al., 2013). The formation and remodeling of myelin is associated with changes in the biochemical features of brain tissue, such as alterations in water, lipids, proteins, and iron content within oligodendrocytes (Alexander et al., 2011). Although white matter plasticity in the human brain has been predominantly studied with diffusion tensor MRI (DT-MRI)-based metrics of fractional anisotropy (FA) or diffusivities (Pierpaoli & Basser, 1996), these indices are not specific to any white matter property and therefore difficult to interpret in terms of biological changes (De Santis, Drakesmith, Bells, Assaf, & Jones, 2014).

In this study, we therefore applied the myelin water fraction (MWF) and the longitudinal relaxation rate R_1_ from the multicomponent-driven equilibrium single-pulse observation of T_1_ and T_2_ (mcDESPOT; Deoni & Kolind, 2015; Deoni, 2011b) as proxy metrics of myelin and the restricted volume fraction (RVF) from the composite hindered and restricted model of diffusion (CHARMED; Assaf & Basser, 2005; Assaf, Freidlin, Rohde, & Basser, 2004) as a proxy metric of axon morphology. These measures were combined with FA for the purposes of comparability with previous training studies that reported increases in FA (Zatorre et al., 2012; Lövdén et al., 2010; Takeuchi, Sekiguchi, et al., 2010; Scholz, Klein, Behrens, & Johansen-Berg, 2009) and with radial diffusivity (RD). Following evidence showing a link between RD and myelin in coaxially aligned fibers in the mouse brain (Song et al., 2005), RD is often interpreted as a metric of myelin despite the inherent problems of interpreting DT-MRI measures in terms of specific biological white matter properties (Wheeler-Kingshott & Cercignani, 2009).

Assuming that white matter plasticity would be driven by myelin-related tissue changes, we hypothesized increases in MWF and R_1_ relaxation time due to reductions in T_1_ and T_2_ components with increasing myelination (Barkhof & van Walderveen, 1999). We also expected training-related increases in RVF due to plasticity-related changes in glia cell morphology (Tavor, Hofstetter, & Assaf, 2013) as well as increased FA and reduced RD, which have previously been linked to myelin plasticity (Sampaio-Baptista et al., 2013; Zatorre et al., 2012). Training-induced microstructural changes were expected in parietofrontal SLF connections, whereas no specific alterations were hypothesized for the cingulum bundle since the extent to which salience network and limbic memory regions may alter with WM trainining remains a matter of debate (Jolles, van Buchem, Crone, & Rombouts, 2013; Rose, Olsen, Craik, & Rosenbaum, 2012). Finally, we explored whether training-induced microstructural changes would correlate with cognitive benefits (Valkanova, Rodriguez, & Ebmeier, 2014).

## Methods

Detailed descriptions of the training procedure and cognitive outcome assessments can be found in Caeyenberghs et al. (2016) and Metzler-Baddeley et al. (2016) and are only briefly summarized here.

### Participants

The study was approved by the Cardiff University School of Psychology ethics committee. Forty-eight healthy adults (19–40 years) participated and gave informed written consent. Participants were randomly allocated to the adaptive training or the comparison group with the provision that both groups were matched for age and sex. Participants were blind to their training condition. Four participants in the training and four in the comparison group dropped out because of time commitments, leaving 20 individuals in each group who completed the study. The two groups were comparable in their demographics and baseline cognitive performance (Table 1).

### WM Training

Participants performed computerized exercises of verbal and spatial span tasks (Cogmed, 2012).^1^ Training was accessed via the Internet from home, and participants had to practice five times per week for 8 weeks (40 training sessions of about 30 hr in total). The number, frequency, and order of training tasks were identical for all participants. Training progress was monitored, and participants received weekly feedback by e-mail. In the adaptive training condition, task difficulty was altered depending on the trainee’s level of performance to ensure that participants exercised at their maximum level of WM capacity. Participants in the comparison group trained on level three item spans throughout all training sessions. Both groups completed the same number of training sessions but, on average, the comparison group spent 7 min less per session because each trial was on average shorter (Table 1).

### Cognitive Assessment

Participants were tested before and after the training with a previously validated battery of computerized assessment tests from Cambridge Brain Sciences (www.cambridgebrainsciences.com; Hampshire, Highfield, Parkin, & Owen, 2012). WM capacity was tested with forward and backward digit span and spatial span, distractor suppression with an adapted version of the Stroop test (Double Trouble), problem solving with a version of the Tower of London task (the Tree task), abstract reasoning with grammatical reasoning and the odd-one-out task, and the ability to manipulate and organize spatial information with a self-ordered spatial span task. Multitasking abilities were tested with the automated symmetry span task (Unsworth, Heitz, Schrock, & Engle, 2005).

### MRI Data Acquisition

MRI data were acquired on a 3-T General Electric HDx MRI system (GE Medical Systems, Milwaukee, WI) using an eight-channel receiver-only head RF coil at the Cardiff University Brain Research Imaging Centre. MRI sessions were interleaved for both groups to avoid confounds between the experimental conditions and any potential scanner-related changes in data acquisition (Thomas & Baker, 2013). T_1_-weighted anatomical FSPGR images (256 × 256 acquisition matrix, repetition time [TR] = 7.8 msec, echo time [TE] = 2.9 msec, flip angle = 20, 172 slices, 1 mm slice thickness, field of view = 23 cm) were acquired. Diffusion data were collected with a spin-echo echo-planar high-angular resolution diffusion imaging (Tuch et al., 2002) sequence with diffusion encoded along 60 isotropically distributed orientations according to an optimized gradient vector scheme (Jones, Horsfield, & Simmons, 1999) and six nondiffusion weighted scans (TR/TE = 87 msec, *b* value = 1200 sec/mm^2^, 60 slices, 96 × 96 acquisition matrix, field of view = 230 × 230 mm, 2.4 mm slice thickness, reconstructed spatial resolution 1.8 × 1.8 × 2.4 mm). Data acquisition was peripherally gated to the cardiac cycle with a total acquisition time of ~30 min. To gain RVF, data were acquired with the CHARMED protocol (TE = 126 msec, TR = 17,000 msec, 45 gradient orientations distributed on four shells, slice thickness = 2.4 mm, maximum *b* value = 8700 sec/mm^2^, spatial resolution 2.4 mm isotropic, acquisition time 13 min). To gain MWF and R_1_ maps, data were acquired with the mcDESPOT protocol (spoiled gradient recalled echo [SPGR] acquisitions: TE = 2.1 msec, TR = 4.7 msec, flip angles = [3°, 4°, 5°, 6°, 7°, 9°, 13°, 18°]; balanced steady-state free precession [bSSFP] acquisitions: TE = 1.6 msec, TR = 3.2 msec, flip angles = [10.6°, 14.1°, 18.5°, 23.8°, 29.1°, 35.3°, 45°, 60°], spatial resolution 1.7 mm isotropic, acquisition time 12 min; Deoni, Rutt, Arun, Pierpaoli, & Jones, 2008). bSSFP acquisitions were repeated with and without 180° RF phase alteration to remove SSFP banding artifacts, and SPGR and IR-SPGR acquisitions were used to correct B_0_- and B_1_-induced errors in the derived MWF estimates (Deoni, 2011a).

### MRI Data Processing

The diffusion-weighted data were corrected for distortions induced by the diffusion-weighted gradients, artifacts due to head motion and EPI-induced geometrical distortions by registering each image volume to the T_1_-weighted anatomical images (Irfanoglu, Walker, Sarlls, Marenco, & Pierpaoli, 2012), with appropriate reorientation of the encoding vectors (Leemans & Jones, 2009) in ExploreDTI (Version 4.8.3; Leemans, Jeurissen, Sijbers, & Jones, 2009). A two compartment model using the free water elimination (FWE) approach (Pasternak, Sochen, Gur, Intrator, & Assaf, 2009) was then fitted to derive maps of (CSF partial volume-corrected) FA and RD in each voxel (Metzler-Baddeley, O’Sullivan, Bells, Pasternak, & Jones, 2012). CHARMED data were corrected for motion and distortion artifacts according to the extrapolation method of Ben-Amitay, Jones, and Assaf (2012) and corrected for CSF partial volume with the FWE approach. The number of distinct fiber populations (1, 2, or 3) in each voxel was obtained using a model selection approach (De Santis et al., 2014) and RVF, that is, the fraction of the signal assigned to restricted diffusion was calculated per voxel with in-house software (De Santis et al., 2014) coded in MATLAB (The MathWorks, Natick, MA).

The SPGR and bSSFP images acquired as part of the mcDESPOT pipeline (Deoni, Rutt, Arun, et al., 2008; Deoni, Rutt, & Jones, 2008) were corrected for motion using the FMRIB Linear Image Registration Tool (Smith, De Stefano, Jenkinson, & Matthews, 2001) to align all images to the first in the acquisition series. The mcDESPOT model was fitted to the data using in-house software coded in C++ (Deoni, Rutt, Arun, et al., 2008; Deoni, Rutt, & Jones, 2008) to obtain maps of MWF and of the intrinsic relaxation times T_1_ and T_2_. All quantitative maps were coregistered to the T_1_-weighted anatomical images. RVF maps were coregistered to the anatomical image using the *Elastix* registration toolbox (Klein, Staring, Murphy, Viergever, & Pluim, 2010) whereas MWF maps (derived from mcDespot) were coregistered using the FMRIB non-linear registration tool FNIRT (Andersson, Jenkinson, & Smith, 2010). Tract-specific MWF indices were corrected for partial volume artifacts by normalizing these values by their tissue volume fraction from the FWE approach (Bells, Deoni, Pasternak, & Jones, 2011).

### Whole-brain Tractography

Whole-brain tractography was performed for each data set using the damped Richardson-Lucy algorithm (Dell’acqua et al., 2010), which was calculated with in-house software coded in MATLAB. The damped Richardson-Lucy tracking algorithm estimated peaks in the fiber orientation density function (fODF) by selecting seed points at the vertices of a 2 × 2 × 2 mm grid superimposed over the image and propagated in 0.5-mm steps along these axes reestimating the fODF peaks at each new location (Jeurissen, Leemans, Jones, Tournier, & Sijbers, 2011). Tracks were terminated if the fODF threshold fell below 0.05 or the direction of pathways changed through an angle greater than 45°. This procedure was then repeated by tracking in the opposite direction from the initial seed point. Three-dimensional fiber reconstructions of the SLF and cingulum subfascicles were achieved by applying waypoint ROI gates (“AND”, “OR,” and “NOT” gates following Boolean logic) to isolate specific tracts from the whole-brain tractography data. ROIs were drawn manually by three operators blind to the experimental group and time of assessment of each data set on color-coded fiber orientation maps in native space following previously validated anatomical landmark protocols (Jones, Christiansen, et al., 2013; Thiebaut de Schotten et al., 2011; Makris et al., 2005). Pairs of baseline and outcome data sets from the different experimental conditions were counterbalanced across the three operators.

### Reconstructions of SLF and Cingulum Bundle Subfascicles

All tracts were reconstructed separately for each hemisphere. The reconstructions of the three SLF subfascicles followed the protocol by Thiebaut de Schotten et al. (2011). A coronal seed ROI was placed at the level of the posterior commissure around the parietal lobe and an axial “NOT” ROI was placed at the level of the lateral sulci around the temporal lobes. SLF1 was located by placing a coronal “AND” ROI at the level with the anterior commissure around the superior frontal gyrus, SLF2 by placing the ROI around the middle frontal gyrus, and SLF3 by drawing the ROI around the inferior frontal gyrus.

The reconstructions of the cingulum fascicles followed the protocol by Jones, Christiansen, et al. (2013). SGC was located by placing two coronal “AND” ROIs: one around the SGC below the corpus callosum (CC) and another one around the cingulum anterior to the midline of the CC (identified on the sagittal plane). The RSC was reconstructed by placing two “AND” ROIs around the cingulum: one coronal ROI posteriorly to the CC midline and one axial “retrosplenial” ROI dorsal to the ventral limit of the splenium. The PHC was located by drawing the “retrosplenial” ROI and a second axial “AND” ROI at the level of the cerebral peduncle. Figure 1 displays reconstructions of the SLF and cingulum subfascicles for one representative data set. Average values of all microstructural metrics were obtained for each white matter tract. The reliability of the metrics derived from the tracts across the three operators was assessed with intraclass correlation coefficient (ICC) for six randomly chosen data sets. MWF, R_1_, and RVF had reliable ICCs of >.8 in all tracts, and FA and RD had ICCs of >.8 for the majority of tracts.

### Statistical Analyses

Statistical analyses were carried out in SPSS Version 20.0 (IBM, 2011). All data were inspected for outliers, defined as values more than three times the standard deviation from the average cognitive or microstructural index for each time point and group. The left SLF1 pathway could not be reconstructed for two adaptive baseline data sets, one adaptive outcome data set, one comparison baseline data set, and three comparison outcome data sets. Right SLF1 subfascicles could not be reconstructed for three baseline and two outcome comparison data sets. Thus, these data were missing from the microstructural analyses. Because of a technical difficulty, four symmetry span data sets were lost during data acquisition (one training and three control). For all remaining data, changes in cognitive and microstructural metrics were calculated for each participant as difference scores between post- and pretraining values.

Because performance measures in WM and executive function tasks have been shown to share underlying cognitive structures (Testa, Bennett, & Ponsford, 2012) and, similarly, microstructural metrics are known to correlate between white matter pathways (Penke et al., 2010), PCA was employed to reduce the complexity of the cognitive change scores in the nine benchmark tests and the 60 microstructural change scores (5 metrics × 6 pathways × 2 hemispheres). PCAs were run on change scores for all participants across both groups. Performance changes in the 11 trained Cogmed tasks were not included in the PCA since due to the nature of the comparison activities (three-item span practice only) all control participants had zero change scores for all trained tasks. Participants in the adaptive training group, however, showed significant improvements in all trained Cogmed tasks (see Figure 3 and Table 3 in Metzler-Baddeley et al., 2016).

Given the relatively small sample size for PCA, we followed recommendations to limit the number of extracted components as much as possible (de Winter, Dodou, & Wieringa, 2009; Preacher & MacCallum, 2002). Choosing the number of components for data summary is always a compromise between selecting too few components that may miss important structures and too many components that reflect noise. Since there is no single recommended method available, we adopted a threefold approach: First, we employed the SPSS default of the Kaiser criterion of including all components with an eigenvalue of >1 (IBM, 2011). Second, we inspected Cattell’s scree plots (Cattell, 1952) to identify the minimal number of components that accounted for most of the variability in the data. Third, we assessed each component with regard to their interpretability. We used a PCA procedure with orthogonal Varimax rotation of the component matrix. Tables 2 and 3 summarize the component loadings for the cognitive and microstructural variables, respectively. Loadings that exceeded a value of 0.5 were considered as significant.

Group differences in the component scores were then assessed with independent *t* tests. Pearson correlation coefficient was calculated between those cognitive and microstructural components that showed significant group differences to assess whether microstructural changes were related to any cognitive benefits of the training.

All statistical tests were corrected for multiple comparison errors with the Bonferroni correction with a family-wise alpha level of 5% (two-tailed) leading to a corrected *p* value of <.0163 for three independent *t* tests on cognitive change, *p* < .0123 for four independent *t* tests on microstructural change, and *p* < .05 for one correlation coefficient.

## Results

### Training-related Changes in Cognition

Three components that accounted for 55% of the variance of performance changes in the cognitive benchmark tests were extracted (Table 2). The first component loaded highly (>0.5) on performance changes in the Stroop, grammatical reasoning, and self-ordered search. Because these tasks all rely on a variety of executive functions including focused attention, distractor suppression, organization, planning, and reasoning, the first component was labeled “executive function” component. The second component loaded on performance changes in the forward digit span, spatial span, and symmetry span task and was therefore labeled “WM capacity” component. The third component loaded on performance changes in backward digit span, the tree task, and the odd-one-out task and was labeled “problem-solving” component.

**Figure 2:**
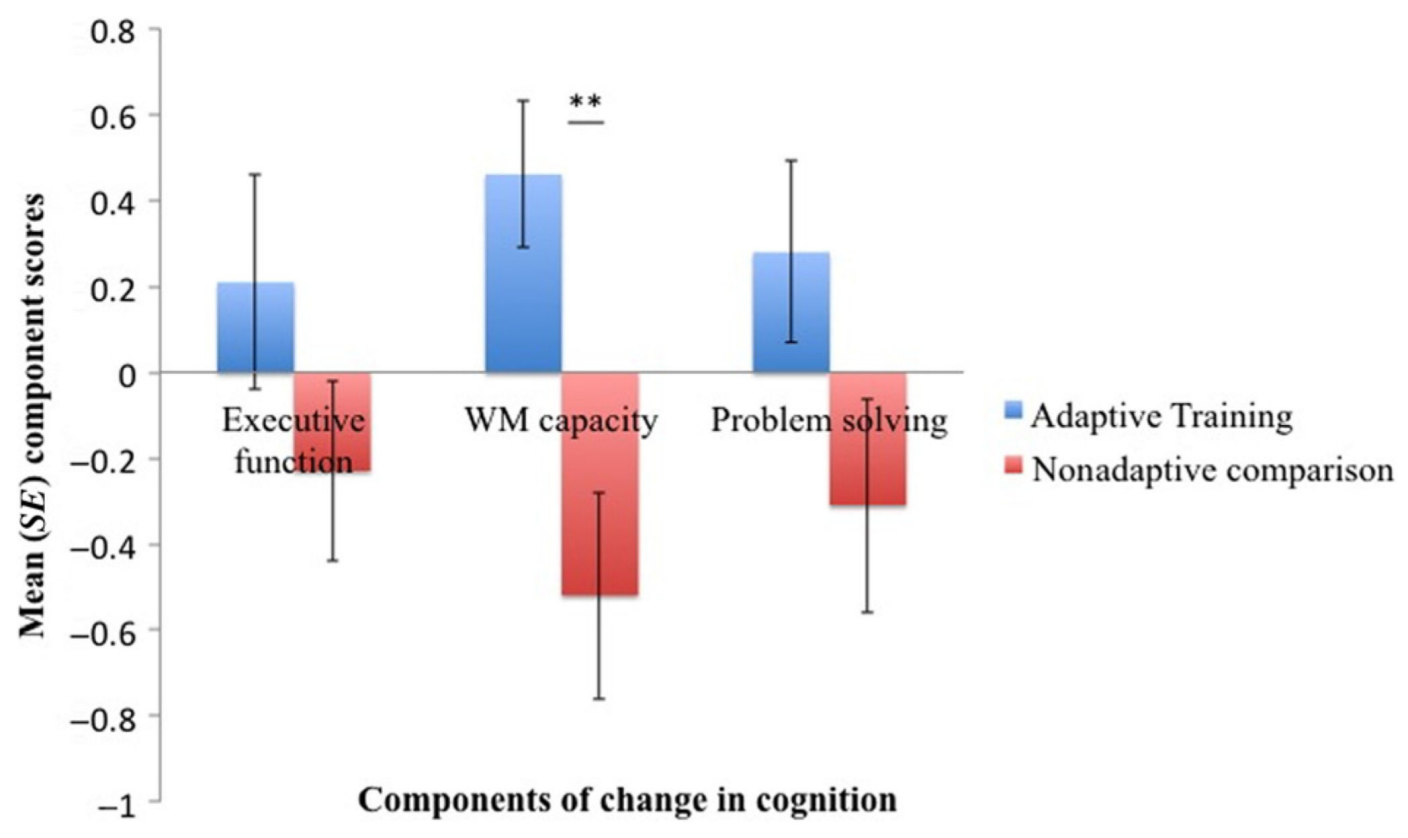
The bar charts display the mean component scores for the “executive function,” “WM capacity,” and “problem-solving” components for the adaptive training group (blue) and the nonadaptive comparison group (red). Components were extracted from change scores of the nine cognitive benchmark tests. The adaptive training group differed significantly from the comparison group in the “WM capacity” component: Adaptive training was associated with positive change, whereas control activities were associated with negative change. No difference was observed for the “executive” and the “problem-solving” components. *SE* = standard error. **p = .002.

To find out if the two groups differed in change in cognition, independent *t* tests were carried out on the scores for the three extracted components. The adaptive training group differed significantly from the comparison group in the WM capacity component scores, *t*(34) = 3.33, *p* = .002, but not in the executive, *t*(34) = 1.3, *p* = .19, or the problem-solving component, *t*(34) = 1.8, *p* = .08 (Figure 2).

### Training-related Changes in White Matter Microstructure

Four components of change in white matter microstructure were extracted, and they explained together 45% of the variability in the data (Table 3). The first component loaded (>0.5) predominantly on change in MWF and R_1_ (“MWF–R_1_” component), the second component on change in the left SLF1 and the left SGC (“left SLF1–SGC” component), the third component on changes in the right SLF1, the left PHC, and the left SLF2 (“right SLF1–left PHC”), and the fourth on changes in the right SGC and right RSC (“right SGC–RSC” component).

**Figure 3:**
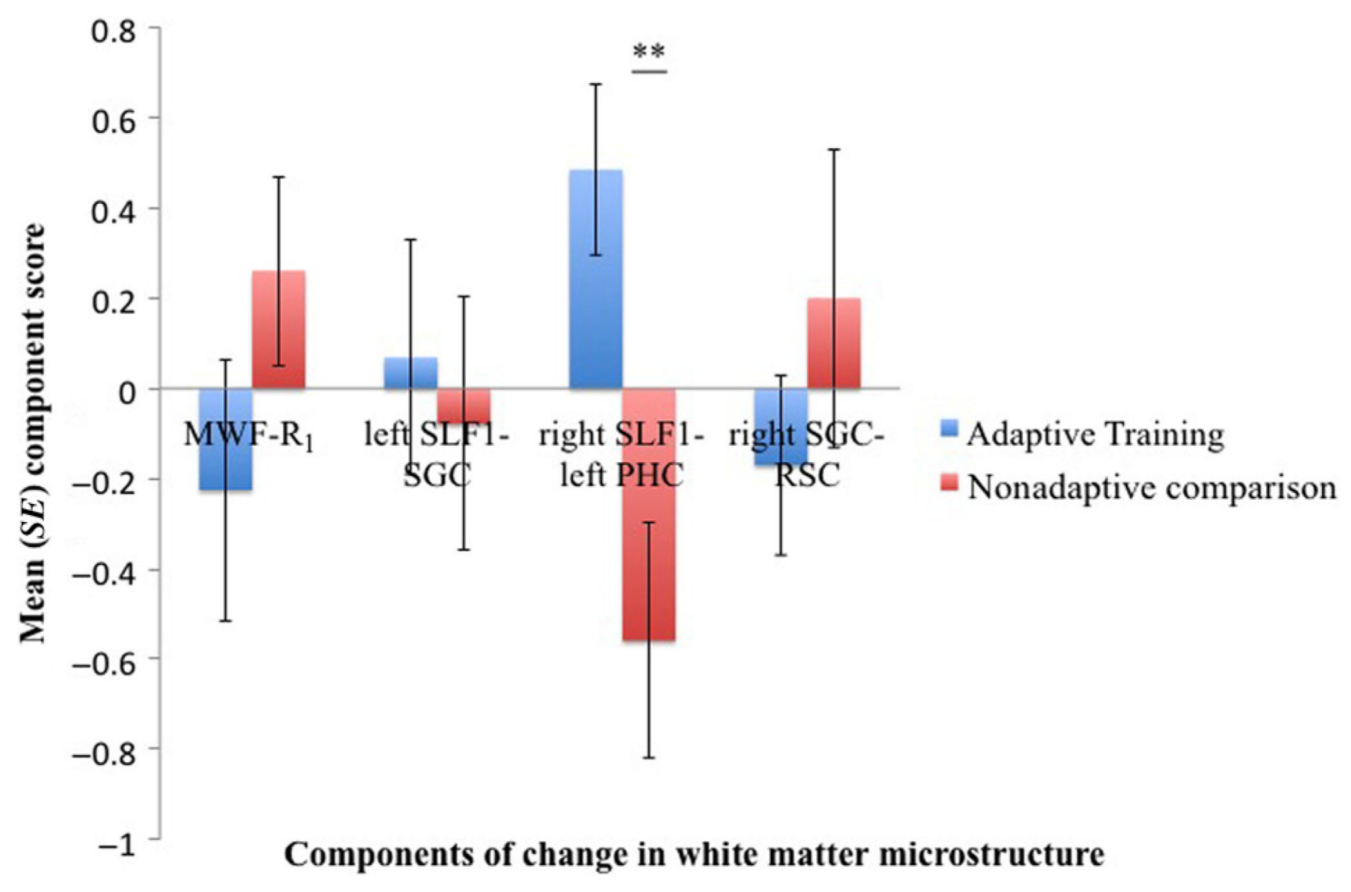
The bar charts display the mean component scores for the four microstructural components extracted from the change scores of average MWF, longitudinal relaxation rate R_1_, RVF, FA, and RD across the SLF and the cingulum bundle. The adaptive training group (blue) differed significantly from the control group (red) in the “right SLF1–left PHC”: Adaptive training was associated with positive change in this component, whereas control activities were associated with negative change. No differences were observed for the other three components. *SE* = standard error. **p = .004.

To assess if there were any group differences in the change in microstructure, independent *t* tests were carried out on the scores for the four extracted components. A significant group difference was present for the “right SLF1–left PHC” component, *t*(26) = 3.2, *p* = .004, but not for any of the other components (MWF–R_1_, *p* = .2; left SLF1–SGC, *p* = .7; right SGC–RSC, *p* = .33; Figure 3).

### Correlation between Cognitive and Microstructural Changes

To ascertain whether changes in microstructure were related to changes in cognition, Pearson correlation coefficient was calculated between the “WM capacity” and the “right SLF1–left PHC network” component scores. No correlation was observed between these component scores (*r* = .1, *p* = .68).

## Discussion

After 2 months of adaptive WM training, participants’ WM capacity was improved compared with comparison volunteers who practiced the same tasks but at a non-challenging level of three items only (Figure 2).

The two groups also differed in a component that loaded highly on microstructural changes in the right SLF1, the left PHC, and the left SLF2. Adaptive training was associated with a positive change in this component, whereas comparison activities were associated with a negative change (Figure 3). From the direction of the component loadings (Table 3), we can infer that adaptive training led to increases in R_1_, RVF, and FA (positive loadings) and to reductions in RD (negative loadings) in the right SLF1 that connects superior parietal with superior and dorsal prefrontal cortical regions (Makris et al., 2005). These changes were in the expected direction and are consistent with previous reports of right-lateralized changes in parietofrontal attention networks (Caeyenberghs et al., 2016; Metzler-Baddeley et al., 2016; Takeuchi, Sekiguchi, et al., 2010; Olesen et al., 2004). We also observed increases in R_1_ and FA and reductions in RD in the left PHC with projections from the posterior cingulate cortex, parietal cortical regions, and the occipital lobe to the medial-temporal lobes (Jones, Christiansen, et al., 2013). It is therefore likely that alterations in the PHC reflect learning, memory, and visual modality-related plastic changes due to the engagement with the adaptive training schedule. In contrast to the PHC, we found no evidence for changes in anterior portions of the cingulum bundle, notably the SGC (with the exception of the “right SLF1-left PHC” component loading on R_1_ in the left RSC), suggesting that the ACC of the salience network was not significantly involved in mediating training effects.

In contrast to the microstructural changes in the right SLF1 and left PHC, we also observed reductions in FA (negative loadings) and increases in RD (positive loading) in the left central parietofrontal white matter of the SLF2. These changes were only observed for FA and RD and are likely the result of differences in fiber complexity and orientation (Jones, Knösche, & Turner, 2013). SLF fibers cross with the corona radiata, an ascending white matter bundle that fans out toward superior cortical regions. Microstructural changes within SLF fibers relative to the corona radiata may therefore have caused opposing effects on diffusion metrics depending on the relative volume fractions of the two white matter pathways. In superior parietofrontal voxels, one would expect the relative volume fraction of the corona radiata to be smaller than the one from the SLF1; hence, training-related increases in microstructural properties of the SLF1 ought to result in increased FA and reduced RD. In contrast in central parietofrontal voxels the relative volume fractions of the SLF2 and the corona radiata may be equal or even larger for the corona radiata; hence, training-related changes in SLF2 fibers might have paradoxically caused a reduction in FA and increases in RD (De Santis et al., 2014). This example highlights the importance of interpreting changes in DT-MRI-based metrics not only in terms of biological white matter properties but also in light of their geometrical and architectural features.

Although fiber complexity-related effects may explain the observed changes in the left SLF2, they cannot account for the opposing group differences in microstructure over time (Figure 3). Figures 2 and 3 demonstrate a clear separation between the two groups: Whereas adaptive training was associated with positive changes in the microstructural and WM capacity component, comparison activities were associated with negative changes in microstructure and cognition.

This pattern of opposing results was unexpected but was observed across a number of modalities. Utilizing morphological data, we recently found similar results of increased cortical thickness for the adaptive training group and reduced thickness for the comparison group in right pFC regions (Metzler-Baddeley et al., 2016) and also observed opposing effects on global efficiency in the parietofrontal network across the groups (Caeyenberghs et al., 2016). We interpret these observations in light of models proposing that activity levels in brain networks are regulated in response to environmental demands with the overall aim to minimize energy consumption (Laughlin & Sejnowski, 2003). Adaptive training may have triggered increased neural and hence axonal activation in parietofrontal WM networks, whereas comparison activities, due to their repetitive and nonchallenge nature, may have resulted in a down-regulation of signaling in these networks since they are not needed for the completion of low-demanding tasks. Evidence from studies into the effects of job demands suggests that prolonged unchallenging activities may adversely affect cognition and brain function. Gajewski et al. (2010) found reductions in WM capacity and electroencephalography in older but not younger assembly line employees compared with age-matched managers. Similarly, Suo et al. (2012) reported that supervisory and managerial experience in midlife was the largest predictor of total gray matter volume in the medial-temporal lobes in a group of older adults. There is a clear need for replicating our findings in a future training study that compares the effects of nonadaptive activities with a passive nonintervention control. Together these results point to the possibility though that the nature of prolonged activities may significantly impact on an individual’s brain structure and cognition.

This study adopted three non-DT-MRI microstructural indices, MWF and R_1_ from mcDESPOT as proxy estimates of axon myelin and RVF from CHARMED as a proxy metric of axon morphology with the aim to find out more about the underpinnings of white matter plasticity. On the basis of accumulating evidence suggesting that axonal activation may trigger biochemical processes in surrounding glia cells that alter water, lipid, protein, and iron concentrations to induce myelination (Fields et al., 2014; Fields, 2010), we expected to find adaptive training-induced increases in MWF, R_1_, RVF, and FA and reductions in RD. Although the expected changes were observed for R_1_, RVF, FA, and RD, we did not find any evidence for training-related changes in the MWF metric that was derived from the two water pool mcDESPOT model. The latter has recently been found to provide insufficiently precise parameter estimates to allow the unambiguous estimation of specific tissue properties such as myelin (Lankford & Does, 2013). In particular, the two-pool model may underestimate MWF in voxels affected by partial volume (Deoni, Matthews, & Kolind, 2013). In this study, DT-MRI and CHARMED indices were corrected for CSF-based partial volume artifacts with the FWE approach by Pasternak et al. (2009), and tract-specific MWF indices were corrected for partial volume by normalizing these values by their tissue volume fraction from the FWE method (Bells et al., 2011). The latter approach may not have sufficiently corrected for partial volume in the MWF metrics. Future studies should therefore apply Deoni et al.’s (2013) novel three-component model that adds to the myelin-associated water pool and the intra/extracellular water pool a third “free water” component to model CSF-based partial volume effects. This three-pool model might provide MWF metrics that are more sensitive to subtle training-induced changes in myelin.

A critical question for all training studies relates to the functional significance of any observed plastic changes. In this study, we did not observe any beneficial effects of WM training on cognitive domains other than specific WM capacity improvements assessed with verbal and spatial span tasks. There is an ongoing debate in the training literature regarding the far transfer effects of WM training (Au et al., 2015; Corbett et al., 2015; Melby-Lervag & Hulme, 2016; Owen et al., 2010), which goes beyond the scope of the current article. Our results suggest that WM training does not lead to generalization effects in healthy adults, but we cannot rule out that transfer effects may be measurable in considerably larger sample sizes (Corbett et al., 2015). In addition, we did not observe a correlation between microstructural and WM capacity component scores. A lack of correlation between structural and functional changes after training has been observed in a number of training studies and may suggest that these processes follow different time courses and may occur in different brain regions (Valkanova, Rodriguez, & Ebmeier, 2013).

In summary, we report activity- and location-dependent plastic changes in the microstructure of parietofrontal and parahippocampal white matter after adaptive versus nonadaptive WM training. Microstructural changes were captured by alterations in R_1_, RVF, FA, and RD and were likely a result of biochemical changes related to myelin remodeling.

## Figures and Tables

**Table 1:** Summary of Demographic Variables and Mean (*SD*) Performance in WM and Executive Function Benchmark Tests of the Two Groups at Baseline

	Training	Controls	t(38)	*p*
*n*	20	20	–	–
Age (years)	26 (6.2)	27 (6.8)	0.44	.67
Female	11	10	–	–
Right-handed	19	20	–	–
Forward digit span	5.3 (0.8)	5.2 (0.7)	0.67	.51
Backward digit span	4 (1.4)	4 (1.4)	0.01	.99
Spatial span	5 (0.5)	4.9 (0.5)	0.97	.34
Stroop (double trouble)	22.8 (13.6)	25.9 (15.4)	0.69	.49
Grammatical reasoning	0.79 (0.2)	0.73 (0.2)	0.97	.34
Tree task	23.7 (8.7)	19.8 (7.2)	0.15	.93
Odd-one-out	9.5 (3.2)	9.1 (4.3)	0.37	.71
Self-ordered search	6.2 (1.1)	5.5 (1.4)	0.18	.07
Symmetry span	25.3 (6.5)	22.6 (7.9)	0.12	.25
Number of training sessions	40	39.9 (0.44)	1.00	.32
Training time per session (min)	42.7 (4.65)	36.31 (6.15)	3.75	.001

**Table 2:** Rotated Component Loadings on Change in the Cognitive Benchmark Tests

Cognitive Change	Executive	WM Capacity	Problem Solving
Forward digit span	−0.017	**0.588**	0.177
Backward digit span	0.230	0.244	**0.677**
Spatial span	0.428	**0.670**	0.167
Stroop (double trouble)	**0.793**	−0.190	0.019
Grammatical reasoning	**−0.746**	−0.278	−0.032
Tree task	0.102	0.220	**−0.801**
Odd-one-out	−0.072	0.470	**0.514**
Self-ordered search	**0.568**	0.068	−0.01
Symmetry span	0.038	**0.653**	−0.24

Loadings >0.5 are highlighted in **bold**.

**Table 3:** Rotated Component Loadings on Change in White Matter Microstructure

		MWF–R_1_	Left SLF1–Left SGC	Right SLF1–Left PHC	Right SGC–RSC
*Myelin Water Fraction (MWF)*					
SGC	L	0.421	0.425	−0.175	0.015
	R	0.483	−0.319	−0.323	**0.506**
RSC	L	**0.767**	0.004	0.129	−0.146
	R	0.303	0.458	−0.025	0.380
PHC	L	**0.624**	0.291	0.337	−0.057
	R	**0.508**	−0.325	0.340	0.024
SLF1	L	0.333	**0.620**	−0.190	−0.117
	R	0.274	0.425	0.243	0.357
SLF2	L	**0.525**	0.175	−0.492	−0.025
	R	**0.624**	−0.107	−0.387	0.142
SLF3	L	**0.733**	−0.001	−0.092	−0.109
	R	**0.654**	−0.045	−0.40	−0.204
					
*Longitudinal Relaxation Rate R_1_*					
SGC	L	0.064	**0.531**	0.125	−0.089
	R	**0.518**	0.086	−0.035	**0.605**
RSC	L	0.304	0.107	**0.526**	−0.127
	R	0.073	**0.512**	0.397	0.363
PHC	L	0.175	0.339	**0.701**	−0.090
	R	0.078	−0.358	0.351	−0.074
SLF1	L	−0.178	**0.689**	−0.034	−0.109
	R	−0.065	0.385	**0.512**	0.379
SLF2	L	0.130	0.265	−0.482	−0.067
	R	**0.583**	−0.069	−0.158	0.136
SLF3	L	**0.525**	−0.034	0.212	−0.193
	R	**0.516**	0.070	−0.115	−0.259
					
*Restricted Volume Fraction (RVF)*					
SGC	L	−0.025	**0.588**	0.127	0.166
	R	−0.199	−0.230	−0.008	**0.533**
RSC	L	**0.509**	0.127	0.121	0.146
	R	−0.072	0.159	−0.221	0.433
PHC	L	0.437	0.336	0.193	0.034
	R	0.150	−0.242	−0.179	−0.028
SLF1	L	−0.116	**0.694**	−0.284	−0.040
	R	−0.111	0.149	**0.611**	0.443
SLF2	L	−0.378	0.487	−0.146	0.300
	R	0.385	−0.023	−0.069	0.190
SLF3	L	−0.047	−0.136	0.323	−0.077
	R	0.229	0.285	−0.263	0.189
					
*Fractional Anisotropy (FA)*					
SGC	L	−0.067	**0.532**	0.055	0.128
	R	0.05	−0.149	0.147	**0.835**
RSC	L	−0.287	−0.176	0.236	−0.186
	R	−0.073	0.044	−0.038	**0.709**
PHC	L	0.15	0.128	**0.577**	−0.127
	R	0.378	−0.47	0.414	−0.017
SLF1	L	0.071	**0.729**	−0.304	−0.136
	R	−0.186	0.31	**0.584**	0.450
SLF2	L	−0.05	0.218	−**0.715**	−0.030
	R	0.211	0.284	−0.281	0.388
SLF3	L	−0.228	0.018	0.264	0.071
	R	0.494	−0.187	−0.062	0.153
					
*Radial Diffusivity (RD)*					
SGC	L	−0.059	−**0.502**	−0.125	−0.025
	R	−0.061	0.189	−0.130	−**0.827**
RSC	L	0.131	0.254	−0.354	−0.059
	R	−0.006	−0.069	−0.164	−**0.603**
PHC	L	0.118	−0.019	−**0.675**	0.030
	R	−0.389	0.527	−0.109	−0.061
SLF1	L	−0.034	−**0.692**	0.374	0.166
	R	0.246	−0.226	−**0.593**	−0.413
SLF2	L	0.042	−0.369	**0.753**	−0.030
	R	−0.317	−0.194	0.287	−0.438
SLF3	L	−0.372	−0.059	−0.094	0.269
	R	−**0.514**	0.172	0.078	−0.131

Loadings >0.5 are highlighted in **bold**.

## Footnotes

1 Cogmed and Cogmed Working Memory Training are trademarks, in the US and/or other countries, of Pearson Education, Inc. or its affiliate(s).
